# Geographical, meteorological and vectorial factors related to malaria re-emergence in Huang-Huai River of central China

**DOI:** 10.1186/1475-2875-9-337

**Published:** 2010-11-24

**Authors:** Shui S Zhou, Fang Huang, Jian J Wang, Shao S Zhang, Yun P Su, Lin H Tang

**Affiliations:** 1 National Institute of Parasitic Diseases, Chinese Center for Disease Control and Prevention; WHO Collaborating Centre for Malaria, Schistosomiasis and Filariasis; Laboratory of Parasite and Vector Biology, Ministry of Health, Shanghai 200025, PR China; 2 Department of Parasitology, Anhui Center for Disease Control and Prevention, Hefei 230061, PR China; 3 Department of Parasitology, Henan Center for Disease Control and Prevention, Zhengzhou, 450003, PR China

## Abstract

**Background**

Malaria still represents a significant public health problem in China, and the cases dramatically increased in the areas along the Huang-Huai River of central China after 2001. Considering spatial aggregation of malaria cases and specific vectors, the geographical, meteorological and vectorial factors were analysed to determine the key factors related to malaria re-emergence in these particular areas.

**Methods**

The geographic information of 357 malaria cases and 603 water bodies in 113 villages were collected to analyse the relationship between the residence of malaria cases and water body. Spearman rank correlation, multiple regression, curve fitting and trend analysis were used to explain the relationship between the meteorological factors and malaria incidence. Entomological investigation was conducted in two sites to get the vectorial capacity and the basic reproductive rate to determine whether the effect of vector lead to malaria re-emergence.

**Results**

The distances from household of cases to the nearest water-body was positive-skew distributed, the median was 60.9 m and 74% malaria cases were inhabited in the extent of 60 m near the water body, and the risk rate of people live there attacked by malaria was higher than others(*OR *= 1.6, 95%*CI *(1.042, 2.463), *P *< 0.05). The annual average temperature and rainfall may have close relationship with annual incidence. The average monthly temperature and rainfall were the key factors, and the correlation coefficients are 0.501 and 0.304(*P *< 0.01), respectively. Moreover, 75.3% changes of monthly malaria incidence contributed to the average monthly temperature (T_mean_), the average temperature of last two months(T_mean01_) and the average rainfall of current month (R_mean_) and the regression equation was Y = -2.085 + 0.839I_1 _+ 0.998T_mean0 _- 0.86T_mean01 _+ 0.16R_mean0_. All the collected mosquitoes were *Anopheles sinensis*. The vectorial capacity and the basic reproductive rate of *An. sinensis *in two sites were 0.6969, 0.4983 and 2.1604, 1.5447, respectively.

**Conclusion**

The spatial distribution between malaria cases and water-body, the changing of meteorological factors, and increasing vectorial capacity and basic reproductive rate of *An. sinensis *leaded to malaria re-emergence in these areas.

## Background

Malaria is an important cause of death and illness in children and adults in tropical countries. According to World Malaria Report 2009[1], half of the world's population is at risk of malaria and an estimated 243 million cases led to nearly 863,000 deaths in 2008. Despite significant reductions in the overall burden of malaria in the 20^th ^century, the disease still represents a significant public health problem in China [2].

Malaria was historically epidemic in the Huang-Huai River region of central China and the total malaria cases in these areas were 21.99 million, accounting for 91.2% of the total reported cases in the country in 1970s. With active implementation of malaria control measures for more than 30 years, considerable success had been achieved and the cases decreased dramatically and many counties in Huang-Huai River region reached the standard of the basic malaria elimination (the incidence is below than 1/10,000). Early in the 21^th ^century, malaria has re-emerged in these areas, especially the Anhui Province, and a total 26,873 malaria cases and 108,594 suspected cases with 23 deaths were reported by the annual case reporting system in 858 counties of 22 Provinces in 2008, and the annual incidence was 0.21/10 000. In central China, the re-emergence of malaria was controlled in 2008, but the number of malaria cases and the incidence in central China still accounts for 68% of the total cases [3]. According to a 2003 national report [4], it was estimated that only 1/18 (5.6%) cases in China were notified and the actual number of cases was higher than reported. Besides, malaria vectors in this area included *Anopheles sinensis *and *Anopheles anthropophagus *historically.

In fact, social and economical status have significantly changed since 1990s in central parts of China, and malaria control interventions also transferred from vectorial controls such as IRS, ITNs combined with case management to enhancing case detection with health education particularly on risk and vulnerable population. Considering the complication and uncertain quality of social and economical factors only the geographical environment and biological factors were analysed to determine the key factors related to the malaria outbreak and re-emergence, which would help to formulate methodologies for malaria monitoring, forecasting and early warning.

## Methods

### Geographical factors

#### Study sites and subjects

**Figure 1:**
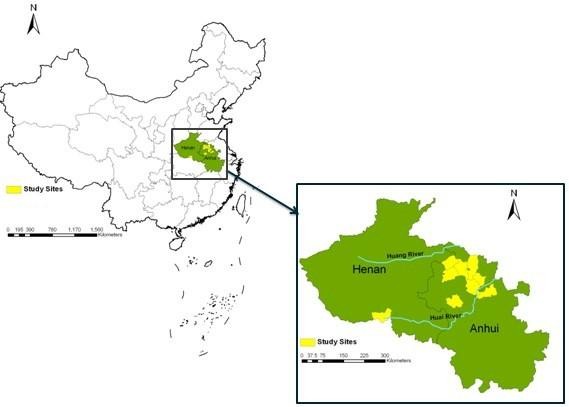
**Study sites for geographical factors analysis**.

According to the identification of the position and the diversity of topographical features, 113 villages from eight counties (Fengyang, Guzhen, Suixi, Guoyang, Yingshang, Yongqiao County in Anhui Province; Yongcheng and Tongbai County in Henan Province) were selected by stratified random unequal proportion sampling method. All the counties were located at 32°17'~34°18' north latitude, 113°~117°09' east longitude along the Huang-Huai River, with malaria re-emergence in recent years (Figure 1).

#### Data collection

The information on malaria cases in eight counties was collected from the national reporting system in 2004~2007, confirmed by a household investigation, conducted to confirm all of the reported malaria cases. The water-body is defined as the paddy fields, gully and little stream, which are suitable breeding sites of *An. sinensis*. The identification of water-bodies was conducted under the supervision of experts, and data on 357 cases households and 603 water-bodies was collected using hand-held GPS (global positioning system) locator.

#### Data analysis

The data of longitude and latitude were imported into Excel. The software of Map source was used to conduct data processing and database was constructed in Excel. Spatial analysis was performed with software ArcGIS 9.2 to calculate the distance between houses of malaria cases and water-body. Buffer analysis was conducted based on multiple buffer rings with water-body to the center. The Straight-Line Distance Function was used to calculate the distances from household of cases to the nearest water-body, based on the results of buffer analysis.

The straight-line distances from the house of cases to the nearest water-body were analysed using software SPSS 11.5. All the residents in an individual village were grouped according to the characteristics of the distribution of malaria cases, and chi-square test was used to analyse the risk of malaria transmission. To exclude the effect of social factors, such as usage of mosquito net and size of family, chi-square test was conducted to identify the difference between groups.

### Meteorological factors

#### Data collection

Monthly climate data including average temperature, average maximum temperature, average minimum temperature, relative humidity and rainfall from 1990 to 2006 in Huaiyuan County and Tongbai County were obtained from China Meteorological Data Sharing Service System [5]. Temperature and rainfall variables are measured in millimeters (mm) and centigrade (°C) respectively. Data on malaria were obtained from the Center for Diseases Prevention and Control of Anhui Province, Henan Province and Diseases Surveillance System of China [4]. Monthly data of the count of blood films examined for malaria and those positive for malaria were obtained from the routinely report by Anhui Province, Henan Province CDC. Data aggregated were available for the years 1990-2006. Population demographics for the county were provided by the Anhui and Henan of Statistics. It was assumed that each resident in the county was at risk for infection of malaria.

#### Spearman rank correlation

The curves of malaria annual incidence, average annual temperature, average annual rainfall and average annual relative humidity were delineated to explain the annual trends of these factors changes and its relationship with the re-emergence of malaria. Considering the cycle of growth and development of *Plasmodium vivax *and anophelines, the new parameters were derived as follow: T_mean01_, T_mean012_, R_mean01_, R_mean012_, M_mean01_, M_mean012_. Spearman correlation analysis was conducted to examine the association between monthly malaria incidence and climate variables using the SPSS software.

### Regressive analysis

#### Curve fitting and trend analysis

Based on the results of the Spearman rank correlation and multiple regression analysis, curve fitting and trend analysis was used to explain the relationship between these factors and malaria transmission.

### Vectorial factors

#### Study site

Huaiyuan County in Anhui Province and Yongcheng County in Henan Province were selected as the study sites for vector investigation. Huaiyuan County is situated at 33°14'north latitude and 116°51'east longitude and Yongcheng County located at 33°48'north latitude and 116°12'east longitude. These were plain landscapes with soybean as the primary crop, and rare paddy fields. Pesticides were seldom used for soybean. Many ditches were distributed around the villages and there was much water in August and September. This water-body was flow relief, clear and shaded, which was a good breeding ground for larvae of *Anopheles*. It was a fixed vectorial area, including *An. sinensis *and *An. anthropophagus *based on historical vectorial investigations in the Huang-Huai River region. The average vector capacity in this area was 0.1686 in 1990s.

#### Adult anopheles collections and morphological identification

To determine the species and numbers of *Anopheles *biting people in the area, collections on human bait, sometimes euphemistically called landing counts, have been performed. Human bait collections were undertaken from 19:30 hours to 00:00 hours. The procedure consisted of sitting down and exposing the legs to host-seeking anopheles. When these landed on any area of the body, and before they started biting then are captured with an oral aspirator (sucking tube), then carefully blown into cardboard cartons covered with mosquito netting. Collectors tired after 3-4 hours, and were best rotated with a fresh team after such an interval. All the collected adult *Anopheles *were identified morphologically.

The entire people involved in human bait were approved by the Ethical Committee of center for China diseases control and prevention.

#### Anopheles biting rate

The total numbers of mosquitoes caught each hour was recorded separately so that the hours of maximum biting are obtained for the different species. All the *Anopheles *in the house and bed nets were collected from 06:00 hours to 07:00 hours and the number of bed nets and people in bed nets were also recorded to calculate the anopheles biting density [6].

#### Blood meal identification

The abdomens of wild-caught, blood-engorged mosquitoes or half-gravid females with some remaining undigested blood were squashed onto filter paper, which was stored in desiccators until tested. The dried blood was extracted subsequently in buffer and tested by ring precipitation reaction [7]. The probability that the mosquito would take a human blood meal during a particular day (*a*) would be obtained based on the human blood index and the number of days the vector lives (*n*). In this study the number of days *An. sinensis *was 2.5 d based on preliminary results [8].

#### Age-grading methods (M)

Determination of the age of female mosquitoes is important for understanding malaria epidemiology and assessing the efficacy of vector control programmes. The method involved an examination of the tracheoles covering the ovaries of unfed females to determine whether a mosquito had or had not laid eggs. The proportion of parous females was obtained. The probability of daily survival of females (an important entomological parameter in malaria epidemiology) was estimated based on the proportion of parous females in the population.

#### Sporogonic (extrinsic) incubation period

The development of both the vector and parasite is temperature dependent. The female *Anopheles *is not immediately infective after taking a blood meal and the parasite requires a period of time within the mosquito for its development to an infective stage. The period is termed the extrinsic incubation period and its duration under favourable conditions of the vector is dependent on ambient temperature and humidity. Optimum conditions for sporogony are between 25°C and 30°C and the duration of sporogonic (extrinsic) development of *P. vivax *in Anopheles in relation to environmental temperature (25°C) was 10 days [9].

#### The vectorial capacity (C) and the basic reproductive rate (Z)

The vectorial capacity is a transmission probability index based on *R*_0_, but incorporates the duration of sporogony, vector density and duration of infectivity in humans sustain malaria, there needs to be a sizeable population of vectors, and their longevity was, therefore, a key factor. In general, vectors tend to die as a result of external factors before completing their full life span, and only a small proportion of mosquitoes survive long enough to transmit disease. The female *Anopheles *takes a blood meal once every 2-4 days. The length between blood meals was dependent on temperature and also on the preference of the vector for biting humans. Sporogony lasts 8-25 days for the most efficient human malaria parasites. This duration is genetically determined and temperature dependent. The average life expectancy of vectors of human malaria is 20-25 days and the average daily death rate is 4-5 per cent. These estimations have been made by direct observation of different anopheline species and are taken into consideration in the determination of the vectorial capacity, which was defined mathematically as:

$$\text{C}=ma^{\text{2}}p^{\text{n}}/-{\text{log}}_{\text{e}}P$$

*R*_0 _combined measures of mosquito infectivity and survival and was calculated using the formula [10]:

$$ma^{\text{2}}p^{\text{n}}/-\text{r}\left({\text{log}}_{\text{e}}P\right)$$

## Results

### Most cases with more risk located in the extent of 60 m far from water-body

**Figure 2:**
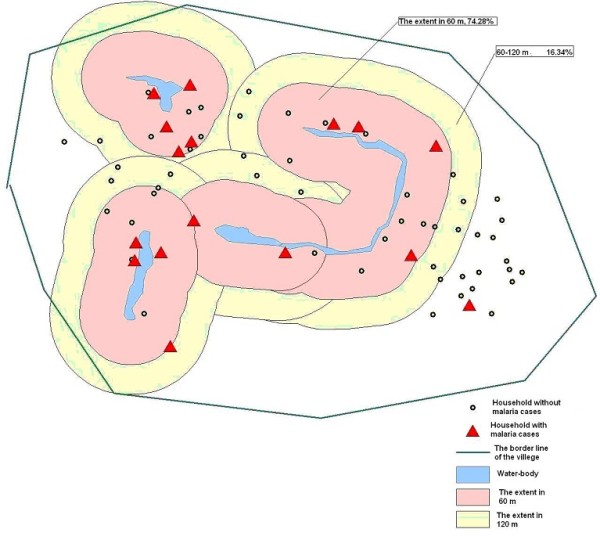
**The proportion of malaria cases in different extent**.

**Figure 3:**
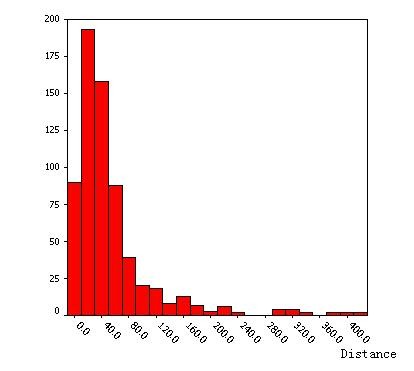
**The statistic distribution of distance from house to water-body**.

The results of analysis in the 90 villages from six counties (Fengyang, Guzhen, Suixi, Guoyang, Yingshang, Yongqiao) showed that the distances from household of cases to the nearest water-body was positive-skew distributed, the median was 60.9 m, which indicated that most cases (74.28%) located in the extent of 60 m far from water-body in villages (Figures 2 and 3).

**Figure 4:**
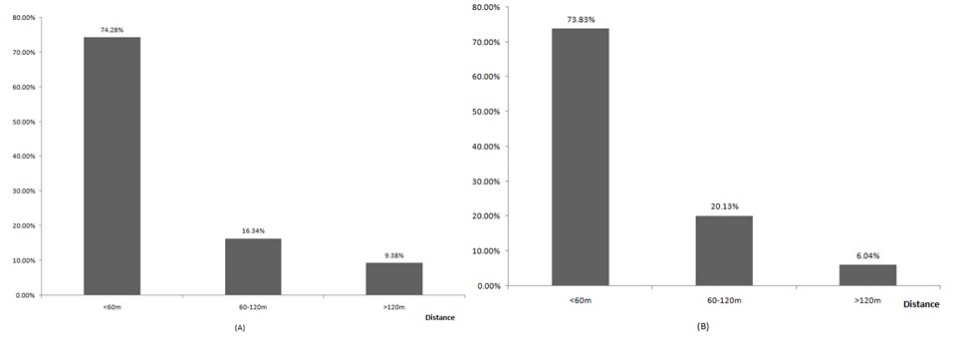
**The percent of malaria cases in different extent**. (A): Anhui Province; (B): Henan Province

Besides, the analysis of the risk scope of malaria transmission was based on the result above. The consistency between Henan and Anhui Province was tested, and comparison results are shown in Figure 4. The residents in each village were divided into two groups (more than 60 m and less than 60 m) by the distance from their houses to the nearest water-body; the proportion of malaria cases in the two groups was calculated through SPSS 11.5. The results showed as follow: χ2 = 4.664, *P *< 0.05; *OR *= 1.602, 95%*CI *(1.042, 2.463), *P *< 0.05. People living in the extent of 60 m near to the water-body had more risk of malaria infection than the farther people, the risk ratio was 1.6.

### Temperature was the key meteorological factors correlation to malaria incidence

**Figure 5:**
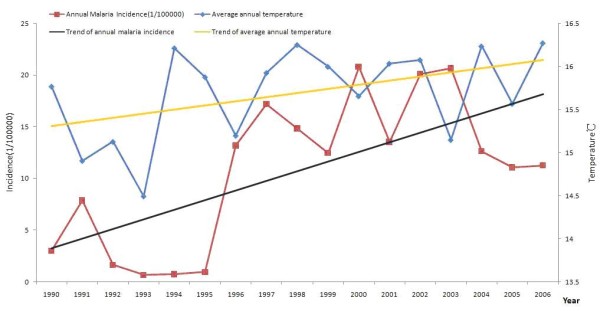
**The trends of average annual temperature and malaria incidence in 1990-2006**.

**Figure 6:**
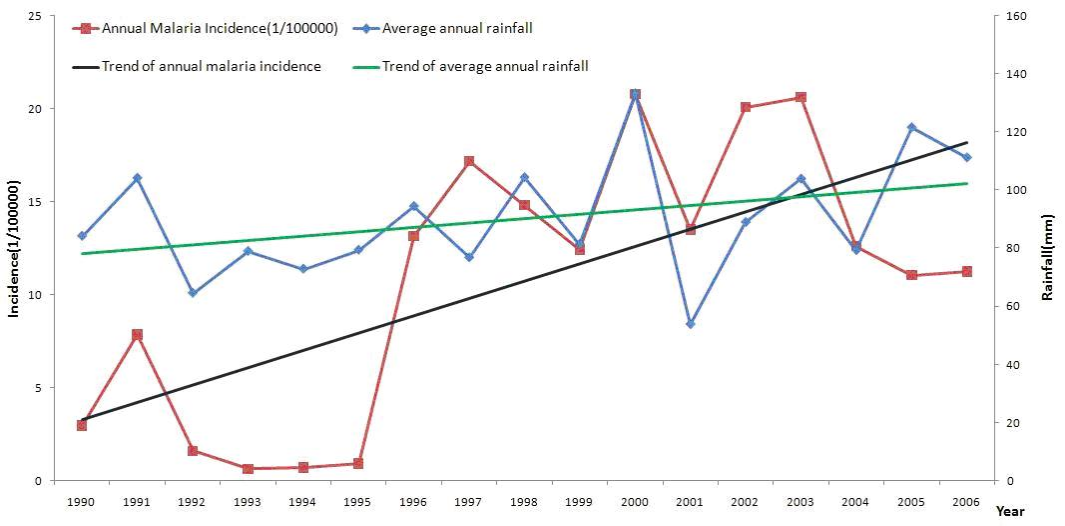
**The trends of average annual rainfall and malaria incidence in 1990-2006, Huaiyuan County**.

The curves of malaria annual incidence, average annual temperature, average annual rainfall and average annual showed that the annual temperature, average annual rainfall may have close relationship with malaria annual incidence (Figures 5 and 6) and relative humid did not. The malaria incidence of Huaiyuan County and Tongbai County increased from 0.33/100,000 to 188.43/100,000, 0.25/100,000 to 637.28/100,000, accordingly the average annual temperature increased 0.87°C and 0.65°C, respectively.

The new parameter was derived based on the meteorological data, including the average temperature of last two months (T_mean01_), the average temperature of last three months (T_mean012_), the average rainfall of last two months (R_mean01_), the average rainfall of last three months (R_mean012_), the relative humidity of last two months (M_mean01_), the relative humidity of last three months (M_mean012_). Spearman correlation showed that T_mean0 _and T_mean01 _were the top two factors with positive correlation to monthly malaria incidence, the correlation coefficients (*r*) were 0.447 and 0.453 (*P *< 0.01), respectively.

Stepwise regression was used to establish the models to determine the contribution rate of all the parameter. Model 4 was the best one with the higher *R*, *R*^2 ^and lower standard error (Table 1 and 2). Therefore, according to Model 4, the regression equation was Y = -2.085 + 0.839I_1 _+ 0.998T_mean0 _- 0.86T_mean01 _+ 0.16R_mean0. _The multiple regression showed that 75.3% changes of monthly malaria incidence contributed to the average monthly temperature (T_mean_), the average temperature of last two months (T_mean01_) and the average rainfall of current month (R_mean_).

**Table 1:** Regression model by stepwise regression

Model	*R*	***R***^***2***^	**Adjusted *R***^***2***^	Standard error
1	.850	.723	.723	11.0025
2	.860	.739	.738	10.6892
3	.865	.749	.747	10.5123
4	.868	.753	.751	10.4356

Note: a. variable in modle1: (Constant), I1;

b. variable in model 2: (Constant), I1, T_mean0_;

c. variable in model 3: (Constant), I1, T_mean0_, T_mean01_;

d. variable in model 4: (Constant), I1, T_mean0_, T_mean01_, R_mean0_;

e. Dependent Variable: I_o_

**Table 2:** Statistical test results of regression model

Model		regression coefficient unstandard (B)	Standard Error of regression coefficient	Standard regression coefficient (β)	*T*	*P*
1	(Constant)	1.511	.567		2.663	.008
	I_1_	.851	.025	.850	34.222	.000
2	(Constant)	-2.951	1.012		-2.916	.004
	I_1_	.817	.025	.817	32.694	.000
	T_mean0_	.303	.058	.131	5.258	.000
3	(Constant)	-1.984	1.024		-1.937	.053
	I_1_	.842	.025	.842	33.218	.000
	T_mean0_	1.076	.200	.466	5.371	.000
	T_mean 01_	-.851	.212	-.355	-4.022	.000
4	(Constant)	-2.085	1.017		-2.049	.041
	I_1_	.839	.025	.839	33.307	.000
	T_mean0_	.998	.201	.432	4.966	.000
	T_mean 01_	-.860	.210	-.359	-4.093	.000
	R_mean0_	.168	.006	.075	2.753	.006

Note: dependent variable I

### The vectorial capacity and the basic reproductive rate were increasing

94 and 107 adult mosquitoes were collected in Huaiyuan County and Yongcheng County, respectively, which were *An. sinensis *by morphological identification. The biting activity, the human biting rate, the vectorial capacity and the basic reproductive rate of *An. sinensis *in Huaiyuan County and Yongcheng County are shown in Table 3. The biting time of *An. sinensis *were from 19:30 to 24:00 locally with a peak time from 21:00 to 24:00. The biting time in Huaiyuan County was earlier than that in Yongcheng County, and the human biting rate, the vectorial capacity and the basic reproductive rate of the former were higher than that of the latter, although the human blood index of the two was similar, which indicated that the transmission capacity of of *An. sinensis *in Huaiyuan County was higher than that of Yongcheng County. Moreover, the vectorial capacities of *An. sinensis *of two sites were 0.6969 and 0.4689, which were 4.12 and 2.78 times higher compared to that of 1990s.

**Table 3:** Vectorial investigation in Huaiyuan and Yongcheng County

Site	No. of adult mosquitoes	The human blood index	Human biting rate	**The vectorial capacity Ma×a×(p**^**n**^**/-lnp)**	The basic reproductive rate*
Huaiyuan County	75	0.6	6.0992	0.6969	2.1604
Yongcheng County	49	0.67	5.1200	0.4983	1.5447

Note: the vectorial capacity of *Anopheles sinensis *was 0.1686 in 1990s.

## Discussion

As with other vector-borne diseases, malaria typically was driven by climatic, ecological and human factors [11-15]. Previous studies [16-18], focused on the *Anopheles flavirostris *and *Anopheles gambiae*, had found a negative relationship between the risk of malaria infection and the distance from the *Anopheles *breeding sites to the houses. Entomological surveys found that the female *Anopheles *requires surface water in which to lay her eggs and in which the larvae hatch after 2-3 days. *Anopheles gambiae *was observed to breed more prolifically in temporary and turbid water bodies, such as those formed by rain, whereas in permanent water bodies predation becomes important. In studies conducted in the area along the Huang and Huai Rivers, *An. sinensis *was the major vector of malaria, with larval habitats in small water-bodies, such as ponds, paddy fields or gullies [19,20]. The results of the present study showed the distances from household of cases to the nearest water-body were positive-skew distributed, the median was 60.9 m and people living in the extent of 60 m near to the water-body had more risk of malaria infection than the farther people. Therefore, it could be suggested to identify the targeted high-risk population and strengthen the treatment to mosquito breeding sites.

Spearman correlation showed that monthly incidence of malaria to various monthly climatic variables and T_mean0 _and T_mean01 _were key factors which had positive correlation to monthly malaria incidence, the correlation coefficients (*r*) were 0.447 and 0.453 (*P *< 0.01), respectively. The multiple regressions showed that 75.3% changes of monthly malaria incidence contributed to T_mean_, T_mean01_, R_mean_. It has been investigated [18-21] that temperature and rainfall played the determinant role of environmental factors in the transmission of malaria Temperature and rainfall may not influence the transmission of malaria in a linear and direct way. The rainfall often leads to small puddles serving as mosquito breeding sites and it increases humidity, which enhances mosquito survival [22,23]. However, the relationship between mosquito abundance and rainfall was non-linear.

The Huang-Huai River region is a stable vectorial area and the severe malaria epidemics in last century were mainly caused by *An. anthropophagus *with higher vectorial capacity. However, malaria outbreaks and re-emergence this time was only in areas with *An. sinensis*, with 94.2% of malaria cases found in this area, which may indicate that the vectorial capacity of *An. sinensis *is increasing. In the past, *An. sinensis *was not an effective transmission vector and malaria incidence absolutely depended on the vectorial capacity and infection source [24]. The vectorial capacities of An. sinensis of two sites were 0.6969 and 0.4689, which were 4.12 and 2.78 times higher compared to that of 1990s [8,25]. Transmission potential may be quantified based upon the basic reproduction rate (*R*_0_) of the parasite. *R*_0 _is defined as the average number of successful offspring that the parasite was intrinsically capable of producing. It was expressed as the average number of secondary infections produced from one infected individual introduced into a non-immune host population. The average basic reproduction rate determines endemicity. For the parasite to survive successfully *R*_0 _must be greater than 1; *R*_0 _values of less than 1 indicate diminishing disease risks and a tendency toward unstable conditions [9]. *R*_0 _values of Huaiyuan County and Yongcheng County were 2.16 and 1.45, respectively, which were greater than 1. In recent years, biological barriers, such as cows and pigs, decreased dramatically, which lead to increasing man-vector contacts and increased transmission rates of *An. sinensis*.

Socio-economic and housing factors also played an important role in malaria transmission, including the presence of open eaves or the lack of ceilings, population density and the presence of animal close to the house, education and available income in household. In this study, these socio-economic factors were not taken into account. Besides, it was acknowledged that there were likely to have been imperfections in the data given that they were obtained from a passive surveillance system. According to a 2003 national report, it was estimated that only 1/18 (5.6%) cases in China were notified [4] and malaria diagnosis was also imperfect, and most of malaria cases were diagnosed based on clinical symptoms.

These findings should be considered in future malaria prevention and control projects, especially in malaria outbreak and re-emergence areas. In 2009, an action plan for malaria elimination was proposed by the Ministry of Health in China. Therefore, it is important for the authorities to know how to distribute limited resources effectively.

## Competing interests

The authors declare that they have no competing interests.

## Authors' contributions

SSZ was responsible for the overall study and the grant from the Ministry of Science, and involved in all stages of this study including design, field work, data analysis and writing manuscript. FH was the focal point in this study and involved in field work and data analysis and wrote the manuscript. SSZ analyzed the relationship between the residence of malaria cases and the water body. JJW and YPS performed to collect malaria incidence data in study sites. All authors read and approved the final manuscript.

## References

[B1] World Malaria Report 2009 Ch4 Geneva, World Health Organization 27 28 http://www.who.int/malaria/world_malaria_report_2009/mal2009_rep_chap4_v2.pdf

[B2] Tang LH Progress in malaria control in China Chin Med J 2000 115 69 92 11775219

[B3] Zhou SS Wang Y Fang W Tang LH Malaria situation in the People's Republic of China in 2008 Chin J Parasitol and Parasit Dis 2009 27 455 45 20232622

[B4] Zhou SS Tang LH Sheng HF Malaria malaria situation in the People's Republic of China in 2003 Chin J Parasitol and Parasit Dis 2005 23 385 387 16566200

[B5] China Meteorological Data Sharing Service System http://cdc.cma.gov.cn/

[B6] Ministry of Health Malaria Surveillance Project in China 2005

[B7] Ministry of Health Disease Prevention and Control Bureau Handbook for malaria control and prevention 2007 Beijing: People's Hygiene Publishing House Press

[B8] Luo MZ Chen CY Studies on the differences of three methods for biting of *Anopheles sinensis *in Wuxue City Chin J Parasit Dis Con 1994 7 219 221

[B9] Qian HL Tang LH Tang LY Zheng ZJ The biting rate and the threshold of vectorial capacity of *Anopheles sinensis* Practical Preventive Medicine 1996 3 1 2

[B10] Warrell David A Gilles Hervert M Essential malariology 2002 London: Hodder Arnold press

[B11] Nihei N Hashida Y Kobayashi M Ishii A Analysis of malaria endemic areas on the Indochina Peninsula using remote sensing Jpn J Infect Dis 2002 55 160 166 12501256

[B12] Klinkenberg E vander Hoek W Amerasinghe FP A malaria risk analysis in an irrigated area in Sri Lanka Acta Trop 2004 89 215 225 10.1016/j.actatropica.2003.08.007 14732243

[B13] Leonardo LR Rivera PT Crisostomo BA Sarol JN Bantayan NC Tiu WU Bergquist NR A study of the environmental determinants of malaria and schistosomiasis in the Philippines using Remote Sensing and Geographic Information Systems Parasitologia 2005 47 105 114 16044679

[B14] Zhou G Sirichaisinthop J Sattabongkot J Jones J Bjornstad ON Yan G Cui L Spatio-temporal distribution of Plasmodium falciparum and P. vivax malaria in Thailand Am J Trop Med Hyg 2005 72 256 262 15772317

[B15] Thomson MC Mason SJ Phindela T Connor SJ Use of rainfall and sea surface temperature monitoring for malaria early warning in Botswana Am J Trop Med Hyg 2005 73 214 221 16014862

[B16] Thomas CJ Lindsay SW Local-scale variation in malaria infection amongst rural Gambian children estimated by satellite remote sensing Trans R Soc Trop Med Hyg 2000 94 159 163 10.1016/S0035-9203(00)90257-8 10897355

[B17] Foley D Torres E Mueller I Stream-bank shade and larval distribution of the Philippine malaria vector Anopheles flavirostris Med Vet Entomol 2002 16 347 335 10.1046/j.1365-2915.2002.00382.x 12510886

[B18] Trape JF Lefebvre-Zante E Legros F Ndiaye G Bouganali H Druilhe P Salem G Vector density gradients and the epidemiology of urban malaria in Dakar, Senegal Am J Trop Med Hyg 1992 47 181 189 1354414 10.4269/ajtmh.1992.47.181

[B19] Gou GX Li DF Shang LY Guo XS Wang WX Sui QL Shen YD Hao JL Hu ZT Liang DP Ding YM The study on ecological habits of *Anopheles sinensis *in Guantang,Luyi county from 1971 to 1996 Chin J Vector Bio Con 1998 9 133 134

[B20] Qu Cz Su Tz Vectorial capacity of malaria transmission of Anopheles sinensis in Zhengzhou in nature Joumal of Henan Medieal University 2000 35 394 396

[B21] Thomson MC Connor SJ A framework for field research in Africa: Malaria early warning systems: concepts, indicators and partners 2001 Geneva, Roll Back Malaria Cabinet Project http://www.rbm.who.int/

[B22] Bi P Tong S Donald K Parton KA Ni J Climatic variables and transmission of malaria: a 12-year data analysis in Shuchen County, China Public Health Rep 2003 118 65 71 10.1016/S0033-3549(04)50218-2 12604766 PMC1497511

[B23] Childs DZ Cattadori IM Suwonkerd W Prajakwong S Boots M Spatiotemporal patterns of malaria incidence in northern Thailand Trans R Soc Trop Med Hyg 2006 100 623 631 10.1016/j.trstmh.2005.09.011 16406037

[B24] Zhao WX Human parasitology 1983 Beijing, People's Hygine Publishing House press

[B25] Hu YX Miao YG Fan TB The further study on ecological habits of *Anopheles sinensis *in the area along Huang River and Huai LRiver Chin J Parasitol Parasit Dis 1988 S1 135

